# Bat Tongues and Foraging: Linking Morphology to Hunting Strategies

**DOI:** 10.1111/1749-4877.12982

**Published:** 2025-04-09

**Authors:** Danilo Russo, Hugo Rebelo, Vanessa Mata, Ana Margarida Augusto, Luca Cistrone, Chiara Belli, Diogo Oliveira

**Affiliations:** ^1^ Laboratory of Animal Ecology and Evolution (AnEcoEvo), Dipartimento di Agraria Università degli Studi di Napoli Federico II Portici Napoli Italy; ^2^ cE3c—Centre for Ecology, Evolution and Environmental Changes & CHANGE—Global Change and Sustainability Institute, Departamento de Biologia Animal, Faculdade de Ciências Universidade de Lisboa Lisboa Portugal; ^3^ CIBIO, Centro de Investigação em Biodiversidade e Recursos Genéticos, InBIO Laboratório Associado, Campus de Vairão Vairão Portugal; ^4^ BIOPOLIS Program in Genomics, Biodiversity and Land Planning, CIBIO, Campus de Vairão Vairão Portugal; ^5^ ICS, Instituto de Ciências Sociais Universidade de Lisboa Lisboa Portugal; ^6^ OnWild—Birding and Photography Experiences Lisboa Portugal

**Keywords:** Chiroptera, ecomorphology, foraging, guild, tongue

## Abstract

On their dorsal surface, bat tongues show a raised muscular structure called mediodorsal lobe (MDL) or lingual prominence. It exhibits different mechanical papillae across species, which we hypothesized are linked to foraging strategies. We predicted that tall MDLs and prominent papillae pointing frontward would effectively trap prey items caught on the wing by aerial hawkers. We examined 904 high‐resolution, close‐up images of 239 individual bats from 24 European species, focusing on MDL height and morphology and arrangement of MDL papillae. Aerial hawkers such as *Tadarida teniotis*, *Nyctalus lasiopterus*, *Miniopterus schreibersii*, and pipistrelles displayed prominent forward‐pointing papillae and taller MDLs, adaptations suited to high‐speed aerial foraging. These traits may be part of a broader “ecomorphological syndrome”, facilitating efficient prey capture in open‐space foragers. In contrast, gleaning and trawling species lacked these specializations, exhibiting flatter MDLs and less prominent papillae. Phylogenetic analysis indicated evolutionary convergence in MDL morphology among aerial hawkers, with *M. schreibersii* showing similarities to vespertilionids despite its phylogenetic distance. This convergence highlights the influence of evolutionary pressures arising from foraging requirements on tongue morphology. Although possessing tall MDLs, rhinolophids are outliers, possibly reflecting their unique perch‐hunting strategy or echolocation. If the patterns we found are confirmed for a larger number of species, MDL morphology could predict foraging style across bat species and be included in future descriptions of foraging guilds.

## Graphical abstract

We linked the bat tongue's mediodorsal lobe (MDL), a muscular prominence, to foraging strategies. Aerial hawkers exhibit tall MDLs and prominent forward‐pointing papillae. The MDL may function as a barrier or filter, preventing unintentional ingestion of non‐food material, aiding in prey handling, and controlling food access during fast flight. Gleaning and trawling bats, using different foraging tactics, have flatter MDLs and less prominent papillae, reflecting distinct ecological adaptations.

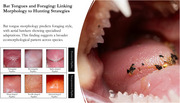

## 1 Introduction

Animal morphology provides a timeless lens into adaptation and evolutionary history, revealing the functions of structures, species roles in ecosystems, and interspecific interactions. In 1862, Charles Darwin hypothesized that an insect with an equally long tongue must have pollinated the deep nectar spur of the Malagasy orchid *Angraecum sesquipedale*. Over a century later, observations in Madagascar confirmed this: Darwin's moth, *Xanthopan morganii praedicta*, emerged as the orchid's exclusive pollinator (Arditti et al. 2012). Even in the digital age, morphology continues to uncover neglected aspects of natural history, with animal tongues offering key insights into feeding adaptations and mutualistic interactions.

The morphology of vertebrate tongues reflects their functional diversity. Composed primarily of muscle, they are often covered with specialized epithelial tissue, including papillae for grip and taste buds for sensory input (Iwasaki 2002; Noel and Hu 2018). Their shape, length, and surface adaptations vary widely, matching species‐specific feeding and ecological roles. The tongue serves as an instrumental organ for capturing, manipulating, and ingesting food and showcases noteworthy morphological diversity in response to environmental factors (Iwasaki 2002; Noel and Hu 2018). Additionally, the tongue aids in grooming and cleaning activities (e.g., Cooper 1994; Mooring et al. 2004; Noel and Hu 2018) and assists in sensory perception through taste receptors on its surface (Kinnamon and Cummings 1992). Thanks to its versatility and adaptability, the tongue is indispensable in many vertebrate species’ feeding and sensory systems. Tongue morphology undergoes specific adaptations in response to the tasks that vertebrates engage in during the manipulation and consumption of food (Noel and Hu 2018).

Bats (order Chiroptera) comprise 1487 species (Simmons and Cirranello 2025) and exhibit diverse diets. Although many bats consume arthropods, their dietary habits extend widely, encompassing frugivory (e.g., Orr et al. 2016), nectarivory (Datzmann et al. 2010), carnivory (Gual‐Suárez and Medellin 2021), piscivory (Aizpurua and Alberdi 2018), and even sanguinivory (Riskin and Carter 2023). Several studies have described the morphology and histology of bat tongues, often focusing on one or a few species (e.g., Pastor et al. 1993; Gregorin 2003; Muchhala 2006; Jackowiak et al. 2009; Park and Lee 2009; Abumandour and El‐Bakary 2013; El‐Mansi et al. 2019; Massoud and Abumandour 2020; Saragih et al. 2020). Detailed structural descriptions of papillae and histological features exist, and these characteristics have been compared among species with different diets (e.g., frugivory vs. insectivory; El‐Mansi et al. 2019). However, cross‐species comparisons within dietary guilds, such as insectivorous bats exhibiting different foraging strategies, remain unexplored.

Nectarivorous phyllostomid bats have evolved specialized tongues for nectar feeding in two subfamilies. Glossophaginae have brush‐tipped tongues, while lonchophyllinae possess grooved tongues that pump nectar (Tschapka et al. 2015). *Anoura fistulata* has an exceptionally long tongue, stored in the thorax when not used (Muchhala 2006). Despite these adaptations being known, ecomorphological studies on bat tongues are scarce, particularly in insectivorous species.

**FIGURE 1:**
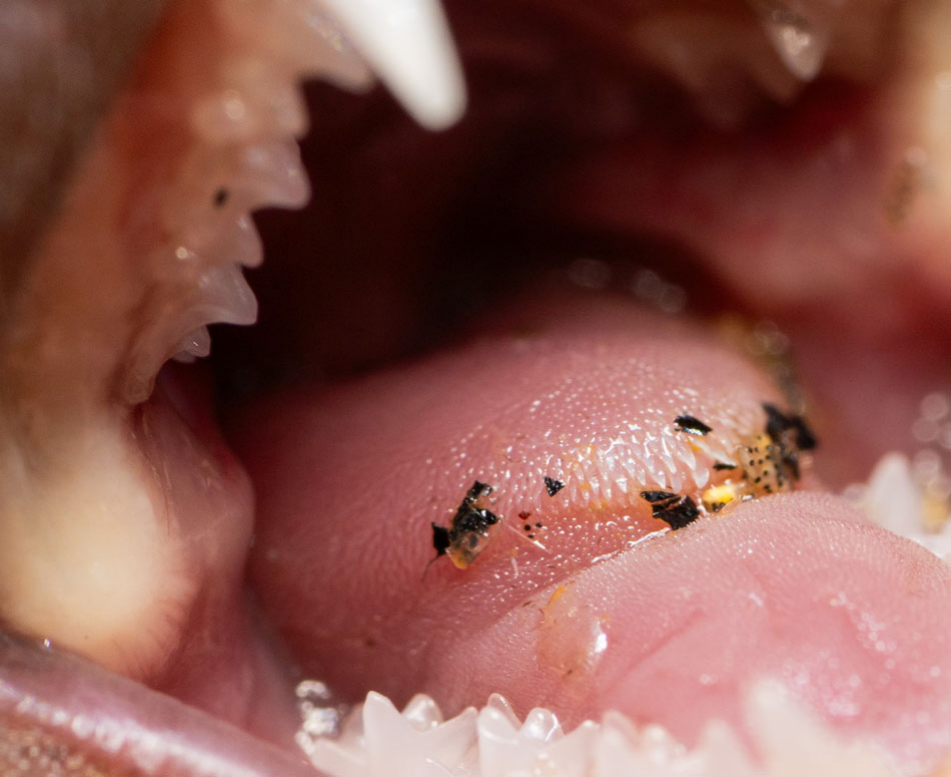
Close‐up of the mediodorsal lobe (MDL) of a greater noctule bat (*Nyctalus lasiopterus*) from Portugal. Note the prominent papillae on the MDL's forefront, which retain exoskeleton fragments of insect prey.

The depiction of general tongue morphology in bats highlights a structure referred to as the mediodorsal lobe (MDL) or lingual prominence, an elevated muscular structure located on the dorsal surface of the tongue, toward the tongue's middle (Figure 1). The MDL function has been likened to that found in numerous herbivorous mammals, such as rodents (Massoud and Abumandour 2020). In such mammals, this muscle‐rich feature, adorned with abundant mechanical papillae, likely plays a pivotal role in the mastication process of cellulose‐rich substances by exerting pressure against the palate. However, the presence of MDL is by no means confined to an herbivorous diet and is widespread in insectivorous bats. The absence of the MDL in pteropodids (Jackowiak et al. 2009; Trzcielińska‐Lorych et al. 2009) suggests that this structure is linked to food handling and processing in insectivorous species rather than being associated with a frugivorous diet (Saragih et al. 2020). However, no descriptions are available for non‐pteropodid frugivorous bats. Based on the few insectivorous species examined so far, the MDL in the Vespertilionidae, like in numerous other families, appears less pronounced than in the Molossidae, where it is markedly developed (Gregorin 2003). Mechanical papillae cover this structure, which likely aids in retaining insects captured by bats using an aerial hawking approach and moving them toward the esophagus (Saragih et al. 2020). Although other studies acknowledge the existence of MDL in bats, none have explored its potential role in food acquisition and manipulation. Particularly, there is a lack of comparative examination across species with diverse diets and foraging techniques.

Bat tongues exhibit various mechanical and gustatory papillae (e.g., Abumandour and El‐Bakary 2013; El‐Mansi et al. 2019; Massoud and Abumandour 2020; Saragih et al. 2020). Within their respective categories, these papillae may display morphological distinctions among species, and the terminology used by different authors is often inconsistent. Typically, bat gustatory papillae consist of (circum)vallate and fungiform papillae; mechanical papillae encompass filiform papillae (which can vary in shape) and conical papillae, which can be small or large and have been regarded as a type of filiform papillae by some authors (e.g., Pastor et al. 1993; Park and Lee 2009). The abundance, distribution, and orientation of these papillae on the tongue's surface can vary among species. Studies have primarily focused on describing papillae and inferring their potential mechanical roles from structural characteristics. In insectivorous bats, mechanical papillae generally predominate over gustatory papillae, likely reflecting their essential function in capturing and manipulating insect prey (Pastor et al. 1993; Sharma et al. 1999; El‐Mansi et al. 2019). Therefore, mechanical papillae likely show differences among different insectivorous bat species in their numbers, size, and orientation in response to prey type and foraging strategy. While potential roles of mechanical papillae in food handling have been proposed (Sharma et al. 1999), this topic requires further investigation.

Insectivorous bats show a broad range of foraging strategies among different species to capture and handle their insect prey, which is well‐matched to their wing structure and echolocation call patterns (Denzinger and Schnitzler 2013; Luo et al. 2019; Zou et al. 2022). Aerial hawkers (e.g., Norberg 1986), for instance, seize their prey directly from the air using their mouths, though some may occasionally use their wings to stop the prey and bring it to their mouth. In contrast, gleaners obtain prey from the ground (Arlettaz et al. 2001) or vegetation (Anderson and Racey 1991) by landing or hovering. On the other hand, trawling bats skim the water's surface, using their larger feet and the tail membrane to gather and transport prey to their mouths (Aizpurua and Alberdi 2018). Consequently, these different strategies present distinct challenges to the predators. While techniques like gleaning or trawling involve a significant degree of control over the prey before consumption, aerial hawking entails a direct mid‐air interaction with the prey. During this interaction, the bats maintain open airways for echolocation and are predicted to have mechanisms to prevent unintentional ingestion of non‐food items or incorrect prey handling. Due to aerial pursuit's high‐speed and dynamic nature, these safeguarding mechanisms might undergo more refinement in bat species that rely on aerial hawking than in those employing other foraging strategies, such as gleaning or trawling. However, no studies have explored how these mechanisms vary among different foraging strategies, leaving a gap in our understanding of their functional significance across varying niches.

Tongue morphology seems to be a well‐suited factor for fulfilling this role. A structure that stops prey caught in flight, especially at high flight speeds, would be essential to assist prey handling. Therefore, the tongues of insectivorous bats are expected to show specific adaptations in high‐speed dynamic foragers, such as well‐developed MDLs and mechanical papillae, to stop the ingestion of non‐food items and secure prey caught in flight. In contrast, such adaptations are not expected in other guilds, such as gleaners or trawlers, where prey is captured at lower speeds. Distinguishing between morpho‐functional adaptation and phylogenetic inertia would be essential (Hansen and Orzack 2005), as certain lingual traits may be conserved among different species due to their shared phylogenetic ancestry rather than being indicative of adaptive responses to a specific foraging strategy. Exploring large datasets encompassing many species is hindered by the threatened status of many bat species and the inevitably invasive approaches, such as euthanizing the bats, that need to be adopted to study tongue morphology in detail. Therefore, non‐invasive ways to study bat morphology are especially welcome, provided they can still offer valuable information despite their limitations (Russo et al. 2017).

In this study, we used non‐invasive macrophotography to examine the size and structure of the MDL in 24 European bat species, analyzing over 900 images. We focused on the mechanical papillae at the forefront of the MDL, as these structures are the first to interact with food items upon entry into the mouth. Image analysis and comparative measurements allowed us to assess potential differences across foraging strategies. We hypothesized that the MDL in aerial hawkers has specific adaptations that are absent in bat species using other foraging strategies. These adaptations likely help prevent the unintentional ingestion of non‐food material and aid in prey handling during fast flight. Specifically, we predicted that aerial hunters would have taller MDLs with more prominent mechanical papillae pointing forward than species with different foraging strategies.

## 2 Materials and Methods

### 2.1 Study Areas

We collected tongue morphology data in mainland Portugal and central Italy in June‐August 2023 (Figure S1). These regions were chosen to ensure comprehensive coverage of as many European bat species as possible. Both study areas host 27 bat species belonging to four different families (Miniopteridae, Molossidae, Rhinolophidae, and Vespertilionidae) and foraging guilds (aerial hawkers, gleaners, perch feeders, and trawlers), enabling us to photograph and characterize the tongue traits of a wide range of bat species.

In Portugal, we sampled the following areas: Serra da Estrela, Manteigas; Serra do Açor, Mata da Margaraça; Sobral da Adiça, Mina da Preguiça; Vimioso, Algoso; Mértola, Ribeira de Carreiras. Serra da Estrela represents Portugal's highest mainland mountain range, towering at 1993 m a.s.l. The region experiences mixed temperate and Mediterranean climates, with diverse vegetation at different elevations, from oak forests to matgrass lawns. Serra do Açor lies in central mainland Portugal, with altitudes up to 1012 m a.s.l. The area has a sub‐humid climate characterized by dry, hot summers and cold, rainy winters. Vegetation includes chestnut and oak trees, along with other notable species. Sobral da Adiça features a significant bat colony in a disused mine. The region has a Mediterranean climate with hot, dry summers and low precipitation, and it is primarily used for agro‐silvo‐pastoral activities. Algoso hosts an important *Tadarida teniotis* Rafinesque colony in a modern bridge. The climate exhibits characteristics lying between meso‐ and supra‐Mediterranean zones, with cold winters and dry, hot summers. The region has plateaus with native evergreen oak woodlands. Ribeira de Carreiras hosts a colony of *Eptesicus isabellinus* (Temminck) in a relatively modern bridge. The climate is Mediterranean with hot dry summers and mild wet winters. Vegetation is dominated by holm oak and rockrose, along with rain‐fed crops.

In Italy, the Abruzzo, Lazio, and Molise National Park and its buffer zone (41°45′46.8″N, 13°58′8.4″E) were sampled for bats over the Sangro River, along an approximately 30‐km stretch. The area's elevational gradient is 800–1100 m a.s.l. Along most of the river's course, well‐established riparian vegetation thrives, mainly characterized by *Salix* spp.

### 2.2 Bat Capture

In Serra da Estrela and Serra do Açor (Portugal), bats were captured at foraging sites using mist nets. To capture bats in roosts, we employed harp traps for cave‐dwelling bats (Sobral da Adiça) and mist nets or bridge traps for crevice‐dwelling species (Amorim and Rebelo 2011; Kingston 2013). In Italy, we selected 26 sites scattered along the river course, where we set up one 6‐m or 12‐m net approximately 30 min after sunset, keeping it active for about 3 h. We recorded the sex and age of each captured bat. We also measured body mass and forearm length (hereafter FAL) with a digital scale (with an accuracy to the nearest 0.1 g) and a caliper (with an accuracy to the nearest 0.1 mm). Species were identified according to Dietz and Kiefer (2016). In both countries, bats were captured under license from the relevant authorities (Portugal, ICNF permits number 274/2023/CAPT, 856/2023/CAPT, and 330/2024/CAPT). In Italy, captures were authorized by the Ministry of the Environment (permit id: PNM_03‐18935_2024‐0111), the Abruzzo Lazio and Molise National Park, and the Abruzzo Regional Government (permit n 0154972/24).

### 2.3 Photography

We photographed the tongue's lateral and frontal aspects of each captured bat using a Sony α7R IV digital camera paired with a Sony 90 mm F2.8 Macro G OSS lens. Lighting was provided by a Sony HVL‐F60RM2 flash at 1/8 power, fitted with a MagSphere MagMod Flash Diffuser for uniform light distribution. A Godox X PRO II Transmitter remotely triggered the flash to ensure optimal alignment with the bat's mouth. The camera was stabilized on a table, allowing the handler to hold the bats while the camera was freely adjusted for the desired angles. The focus was set to manual, at the closest possible distance for 1:1 macro photography, approximately 15 cm from the bat's head. The camera was set to manual mode with ISO 200, f/11, and a shutter speed of 1/250 s. Images were saved as RAW files and later edited in Adobe Lightroom to adjust color and lighting as needed. All images were captured in the field at night, shortly before releasing the bats. Every effort was made to minimize stress and invasiveness during the process. The photographs were taken in a controlled manner at each site to ensure consistency, adopting the same setup (complete darkness except for flash illumination and the same distance from the subject) to maintain consistent photographic settings and handling protocols across all sites and reduce potential biases.

### 2.4 Image Processing

We used ImageJ, a public domain software, to process and analyze the images (Schneider et al. 2012). We described the morphology of the conical papillae in the MDL's forefront section based on the papilla's shape and prominence. Since the existing literature lacks a detailed and consistent description of the papilla shape, we developed a new classification to emphasize the potential adaptive value of papilla shape in prey handling and provide a “taxonomy” based on such characteristics.

**FIGURE 2:**
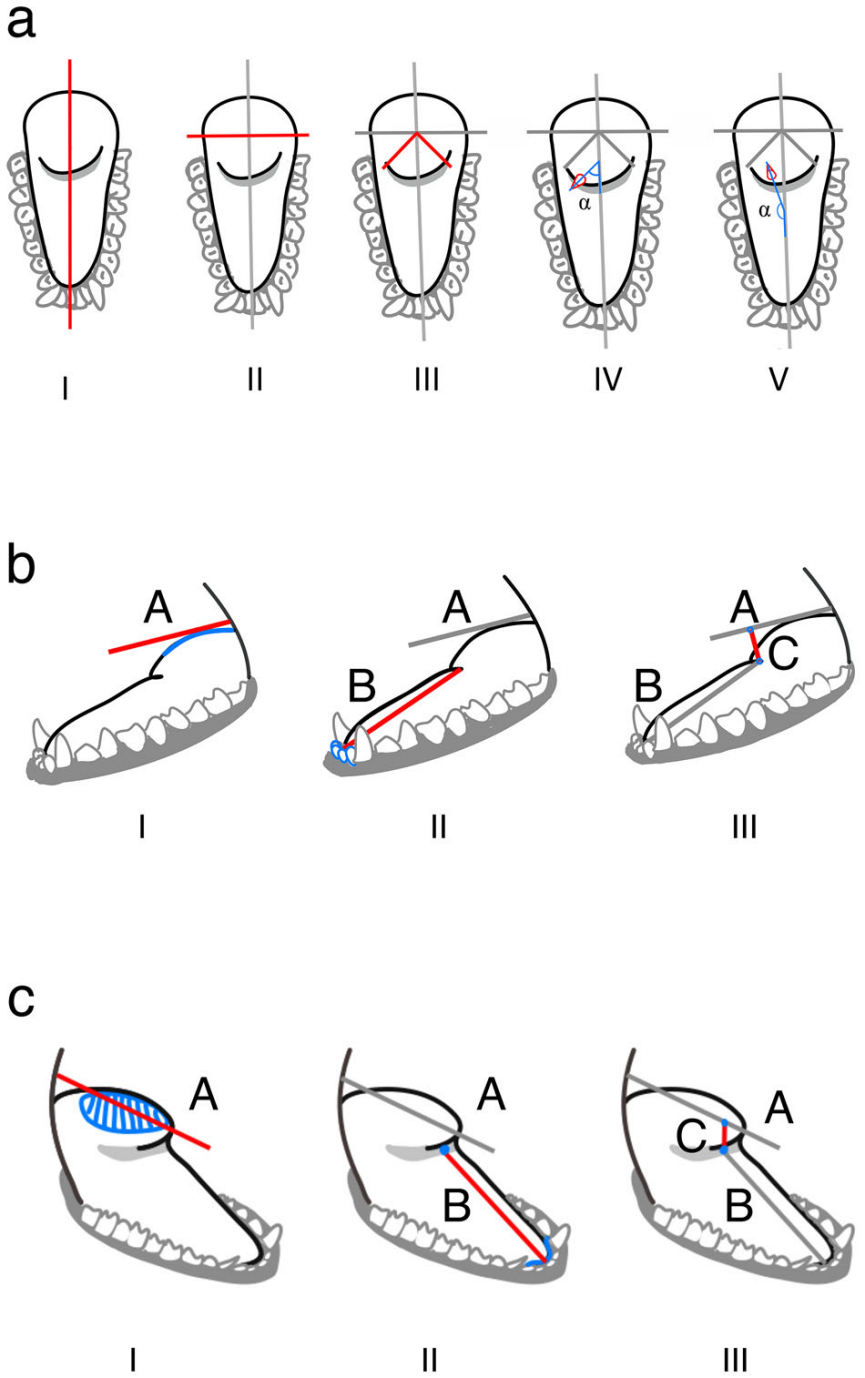
Procedure followed to estimate the orientation (α) and height of bat tongue's mediodorsal lobe MDL (C). Steps I–V illustrate the measurement of α in 10 randomly selected mechanical papillae; IV and V demonstrate how the angle was measured when papillae pointed forward or backward, respectively. The drop‐like silhouette in IV and V provides a schematic representation of the papillae. (b,c) Steps I–III show the measurement of MDL height (C) using tongue images in a lateral perspective (a) or slightly shifted from the optimal perspective (b). The C value was expressed relative to B, with B set to 1.

To measure the angles of the papillae on the lingual prominence, we analyzed images showing the animal's tongue in a frontal position. All analyzed papillae belonged to the “conical” group, the only type found in the MDL. We divided the tongue into four sectors using a cross grid, focusing on the two medial sectors corresponding to the part of the MDL most exposed to incoming food items. To create the grid, we began by drawing a straight line along the central axis of the tongue, dividing it longitudinally, and extending the line across the entire length of the tongue (Figure 2a). Next, we drew a second, horizontal line perpendicular to the first one, dividing the lingual prominence into two equal sections. We then added two lines in the lower quadrants to define the area for measuring the papillary angles. These lines served as bisectors of the lower sectors, creating two angles of 45° each, with one angle open to the right and the other to the left (Figure 2a).

We randomly selected five conical papillae from each side (right and left) within these sections and measured the angles of 10 papillae per individual. To determine the orientation of each papilla, we calculated the angle between the line passing through the longitudinal center of the tongue and the bisector of the papilla (Figure 2a). Angles <90° indicated papillae pointing frontward; angles >90° corresponded to papillae pointing backward. In most cases, at least two images per individual were analyzed. We reduced the number of photos only if fewer than two images were available or their quality was insufficient for accurate analysis.

To measure the height of the lingual prominence, we selected images that showed the tongue in a lateral perspective whenever possible. Two straight lines were drawn as guidelines for these measurements (Figure 2b). The first line (A) was drawn tangent to the upper limit of the prominence, while the second line (B) extended from the base of the prominence to the space between the two medial incisors. Line B served as a reference scale, with its length set to 1 unit, enabling us to obtain a relative value for line C. We drew line C from the base of the lingual prominence to where it intersected line A, following the shortest possible path.

In the few cases when lateral perspective images were unavailable, we used a three‐dimensional approach with images slightly shifted from the ideal perspective (Figure 2c). In these cases, we drew line A to touch the geometric center of the upper part of the prominence and positioned line B along the central axis of the tongue, extending it to the teeth. To establish the MDL's geometric center, we fitted a circle around its limits and identified the center, which coincided with the circle's center (see Figure 2c–I). The procedure for measuring line C remained consistent. This method helped minimize errors caused by variations in image perspective. As with the angle measurements, we analyzed at least two images per individual. The number of photos was reduced only if fewer than two images were available or their quality was insufficient.

### 2.5 Statistical Analysis

We compared the MDL morphology among the different bat species and foraging guilds using three trait variables, one categorical (shape of the papillae) and two continuous (estimated papilla angle and MDL height). We used a tree of all extant mammals built by Faurby and Svenning (2015) to account for potential phylogenetic effects. Because some foraging guilds had few representative species (*N* < 3 for trawlers and perch feeders), statistical analyses were performed only for aerial hawkers (*N* = 13) and gleaners (*N* = 7), and descriptive plots were built including all taxa.

Phylogenetic generalized least squares (PGLS) models with Pagel's lambda (λ) correlation structure were used to test whether the estimated papilla angle and estimated MDL height differed between foraging guilds, and FAL (in mm) was included as a covariate to account for potential size‐related differences among bats. The “gls” function from the “nlme” package was used, with a separate model built for each trait, setting the correlation structure to “corPagel” and using the published phylogenetic tree.

To further explore and visualize the impact of the foraging guild on MDL morphology, we applied a factor analysis of mixed data (FAMD). This analysis combines the principles of principal component analysis (PCA) for quantitative data and multiple correspondence analysis (MCA) for categorical data. It provides a reduced‐dimensional data representation, illustrating how the three variables contribute to the principal axes. PGLS was used as in the previous case, but this time to remove the phylogenetic signal from each continuous trait variable. For this, a null model (trait∼1) was built for each continuous trait, and the residuals were extracted to obtain trait values independent of phylogenetic relatedness. We then used the function “FAMD” from the package “FactoMineR” and the functions “get_famd_ind” and “get_famd_var” to extract dimensional coordinates for bat species and traits.

## 3 Results

**FIGURE 3:**
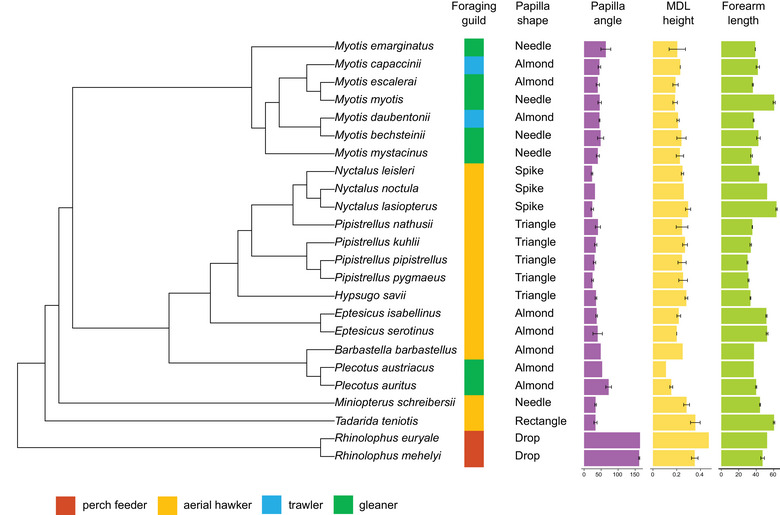
Phylogenetic tree showing all bat species analyzed, their foraging guilds, papilla shape, and the average and 95% confidence intervals for estimated papilla angle, medio‐dorsal lobe (MDL) height, and forearm length (FAL).

**FIGURE 4:**
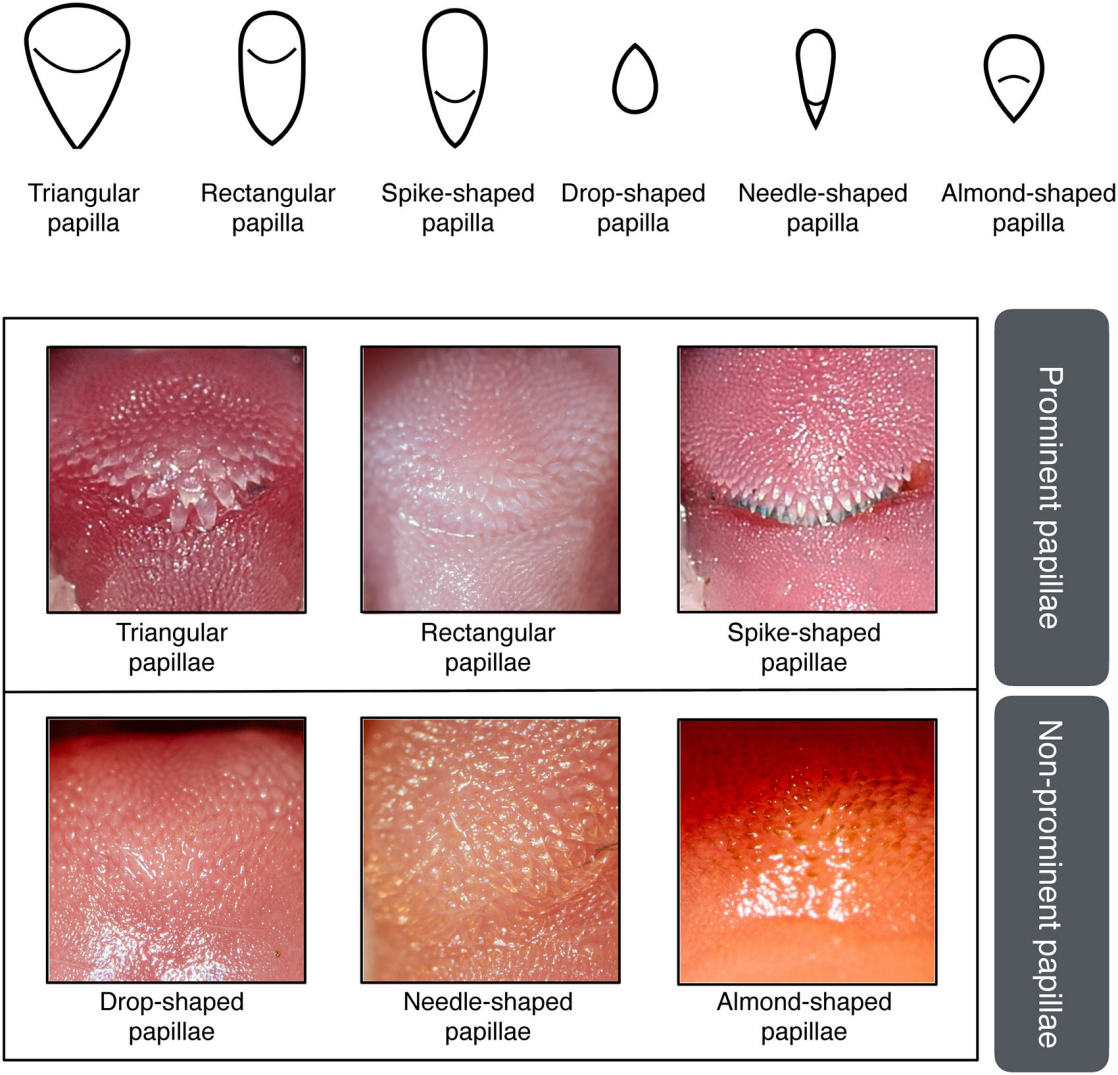
Classification and examples of mechanical papillae observed in the distal section of the tongue's mediodorsal lobe in 24 European bat species. The classification scheme illustrates the diversity of papilla shapes, while representative examples are shown for six species. From the top left corner, reading across the upper row: *Pipistrellus kuhlii, Tadarida teniotis*, and *Nyctalus lasiopterus*. In the lower row: *Rhinolophus mehelyi, Myotis myotis*, and *Plecotus auritus*. Mechanical papillae in these species measure 0.1–0.2 mm (e.g., Pastor et al. 1993; Park and Lee 2009; Massoud and Abumandour 2020).

We examined 904 images of 239 individual bats from 24 species (Figure 3). We obtained a morphological classification of mechanical papillae observed on the MDL, with special reference to its distal section (Figure 4).

### 3.1 Papillae Morphology

We identified two main categories of mechanical papillae on the MDL's frontal area: prominent papillae, which give the MDL a textured, uneven surface, and non‐prominent papillae, which result in a smoother, more uniform appearance (Figure 4). Prominent papillae include triangular, rectangular, and spike‐shaped papillae, while non‐prominent papillae comprise drop‐shaped, needle‐shaped, and almond‐shaped papillae.

1. Triangular papillae (Figure 4) are significantly larger (more than twice the size) than surrounding papillae, creating a distinctly uneven surface on the MDL. They narrow from a broad base to a pointed tip, forming a triangular shape. Arranged in a triangular pattern with the largest papillae positioned toward the front, they are typical the genera *Pipistrellus* and *Hypsugo*.
2. Rectangular papillae (Figure 4) are slightly smaller than triangular papillae. These papillae maintain a consistent width from base to tip, with minimal thinning at the end, resulting in a rectangular appearance. They are arranged in a triangle, with the largest papillae oriented downward. This type is found in the genus *Tadarida*.
3. Spike‐shaped papillae (Figure 4) are markedly larger (more than twice the size) than their neighbors, giving the MDL a pronounced unevenness. They retain their width from the base to halfway along their length, then taper to a sharp point, resembling spikes or a U‐shape. Arranged in semicircular rows like eyelashes on an eyelid, they are present in the genus *Nyctalus*.
4. Drop‐shaped papillae (Figure 4) are similar in size to surrounding papillae, resulting in a uniform MDL surface. They are directed inward, toward the mouth, forming a drop or tear shape with a broad base and tapered tip. Tips may be bifurcated or trifurcated. This type is characteristic of the genus *Rhinolophus*.
5. Needle‐shaped papillae (Figure 4) are thin, elongated papillae that do not differ significantly in size from their surroundings, maintaining a uniform surface. Unlike drop‐shaped papillae, they are mostly oriented downward, especially in the lower MDL. Their shape is needle‐like, tapering gradually from the base to a long, pointed tip. Such papillae are found in *Miniopterus schreibersii* (Kuhl), *Myotis bechsteinii* (Kuhl), *Myotis emarginatus* (É. Geoffroy Saint‐Hilaire), *Myotis myotis* (Borkhausen), and *Myotis mystacinus* (Kuhl).
6. Finally, almond‐shaped papillae (Figure 4) are relatively uniform in size, contributing to a smooth MDL surface. They are not all directed inward but often have a sideways or downward orientation, especially in the central and lower MDL. They have a rounded, flat base that quickly tapers to a tip, resembling an almond. This type is observed in the genus *Plecotus* É. Geoffroy Saint‐Hilaire., in *Barbastella barbastellus* (Schreber), *Myotis capaccinii* (Bonaparte), *Myotis daubentonii* (Kuhl), *Myotis escalerai* Cabrera, *E. isabellinus*, and *Eptesicus serotinus* (Schreber).

Aerial hawkers generally had prominent papillae, except for *Eptesicus* spp., *B. barbastellus*, and *M. schreibersii*. All other specialized aerial hawkers confirmed our prediction of prominent papillae (Figure 4).

### 3.2 Quantitative Patterns in Species and Foraging Guilds

**FIGURE 5:**
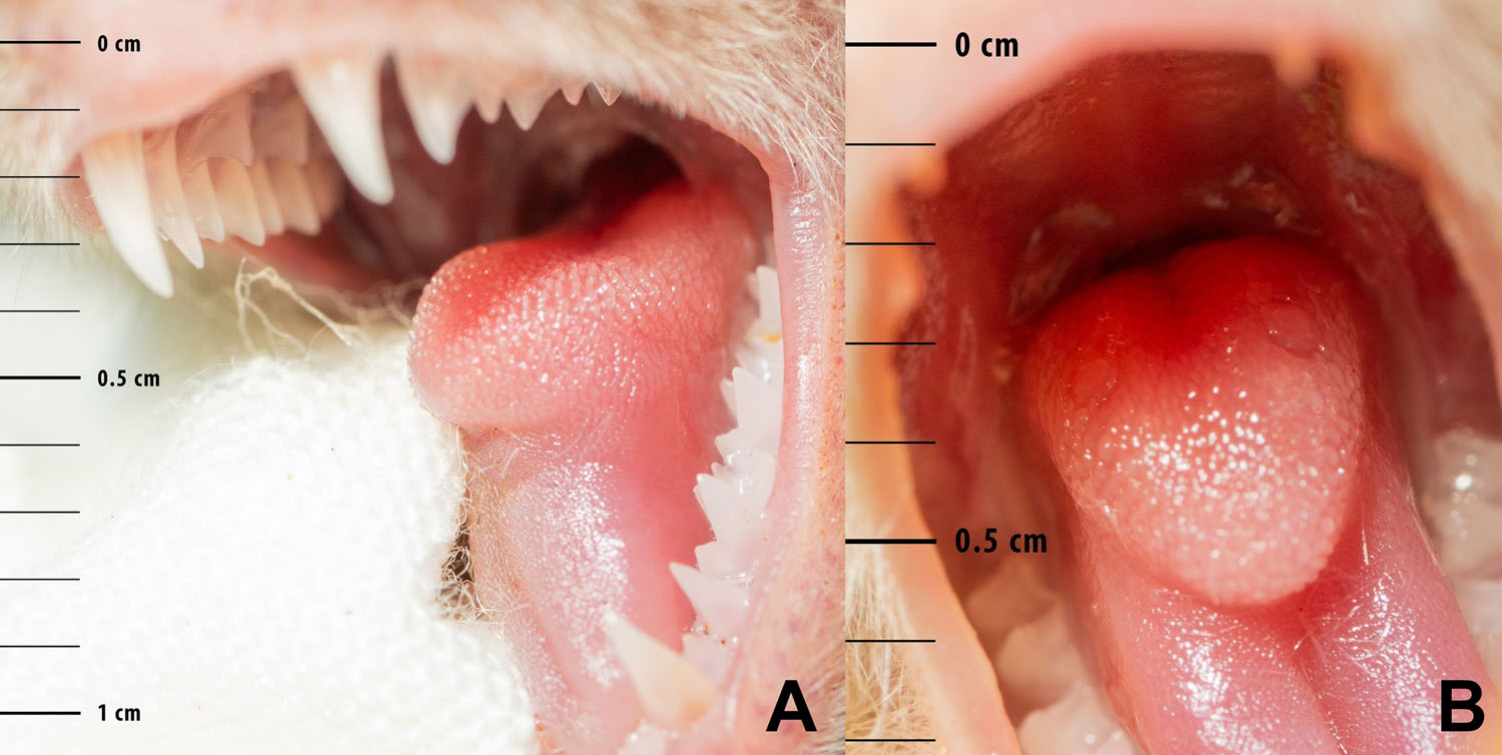
Protrusion of the mediodorsal lobe in hand‐held rhinolophids: *Rhinolophus euryale* (A) and *Rhinolophus mehelyi* (B).

Besides exhibiting a unique drop‐shaped papilla (Figure 4), rhinolophids stood out due to their inward‐pointing papilla (very high estimated papilla angle) and exceptionally tall MDL (“perch feeders” in Figure S2; Figure 3) which could protrude and contract much more than in the other bat families we examined (e.g., Figure 5). MDL was also tall in most aerial hawkers (except for *Eptesicus* spp.), especially *T. teniotis* (Figure 3; Figure S2).

The PGLS analysis showed a significant difference in estimated papilla angle and MDL height between aerial hawking and gleaning species (*p* = 0.0255 and *p* = 0.0045, respectively; Table 1), with no effect of bat size on these variables (Table 1).

**TABLE 1:** Phylogenetic generalized least squares (PGLS) models with Pagel's lambda (λ) correlation structure examining the effect of guild (aerial hawkers vs. gleaners) and forearm length (FAL) on estimated papilla angle and mediodorsal lobe (MDL) height. Significant *p* values are highlighted in bold. Sample size: 13 aerial hawkers and 7 gleaners.

Model	lambda	Coefficient	Estimate	SE	*t*‐value	*p* value
angle ∼ guild + FAL	0.8915					
		(Intercept)	37.751	11.620	3.249	**0.0047**
		guildgleaner	18.075	7.382	2.448	**0.0255**
		FAL	−0.058	0.186	−0.312	0.7592
height ∼ guild + FAL	0.9673					
		(Intercept)	0.262	0.043	6.156	**0.0000**
		guildgleaner	−0.094	0.029	−3.268	**0.0045**
		FAL	0.001	0.001	1.118	0.2793

**FIGURE 6:**
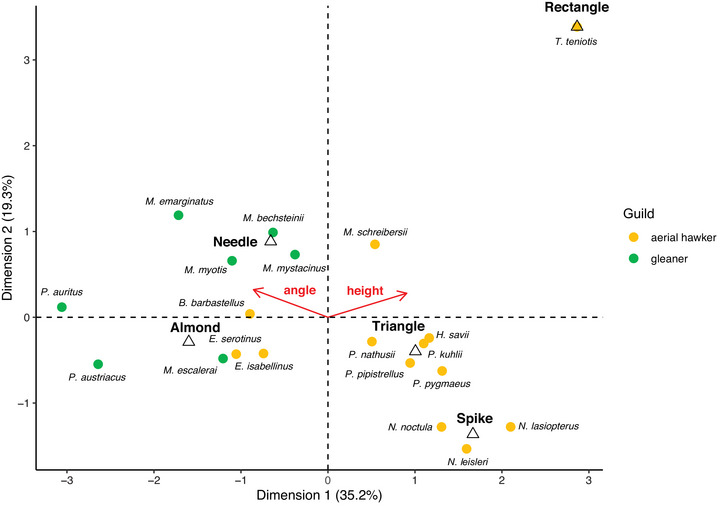
Bidimensional representation of FAMD on three traits of the tongue's mediodorsal lobe (MDL) of 20 European bat species (shape—triangles—and angle of mechanical papillae and estimated MDL height—arrows). Results are based on the first two components representing most of the variance.

The FAMD results (Figure 6) divided aerial hawking species from gleaners, confirming our prediction. The first component was strongly influenced by the papilla's shape, angle, and estimated MDL height (each contributing > 25%). The second component was dominated by shape (over 80%), with the other variables contributing negligibly (<10%). The first component had higher values for decreasing estimated papilla angles and increasing estimated MDL heights. Consequently, species on the right half of the graph (all aerial hawkers) share frontward‐pointing papillae and taller MDLs, as predicted for this foraging strategy. *M. schreibersii* (the only member of the family Miniopteridae included in the analysis) also had a tall MDL and frontward‐pointing papillae. Due to the species’ needle‐shaped papillae, it falls outside the aerial hawker cluster, though still on the right half of the graph (Figure 6).

**FIGURE 7:**
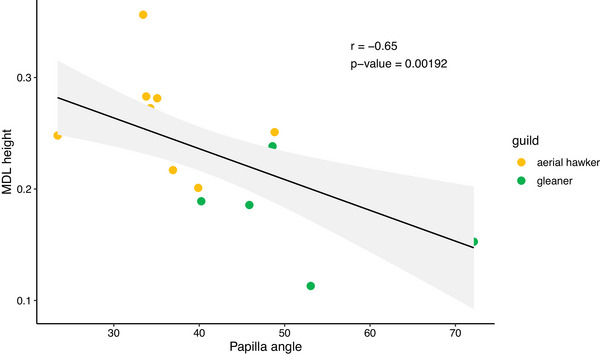
Linear regression between the papilla aspect relative to the tongue's major axis and the relative height of the mediodorsal lobe in 20 European bat species.

The relationship between estimated MDL height and the angle between the papillae and the tongue's major axis corroborates our first hypothesis, with aerial hawkers characterized by tall MDLs and papillae pointing frontward (Figure 7).

## 4 Discussion

In support of our hypothesis, we found that aerial hawkers exhibit more pronounced forward‐pointing papillae and taller MDLs, forming an “ecomorphological syndrome” (i.e., a set of related morphological traits consistently associated with specific ecological roles). The MDL is particularly tall in fast open‐space foraging bats, such as *T. teniotis*, *Nyctalus lasiopterus* (Schreber), and *M. schreibersii*. *Tadarida teniotis* can attain ground speeds up to 149 km h^−1^ (O'Mara et al. 2021). The other two species also reach high speeds in open space (Norberg and Rayner 1987), where they pursue prey such as insects (Aihartza et al. 2023), and, in the case of *N. lasiopterus*, migrating birds during the autumn migration peak (Ibáñez et al. 2016). The findings highlight an important aspect of evolutionary convergence in bats. Different species have evolved similar morphological traits across distant lineages in response to analogous ecological pressures. In the case of *M. schreibersii*, despite phylogenetic divergence, the functional demands of high‐speed aerial foraging have resulted in an MDL structure reminding that of vespertilionid aerial hawkers. This convergence underscores the selective power of foraging niche specialization, especially in open‐space foragers. However, when all three variables were considered in the analysis after phylogenetic correction, *M. schreibersii* diverged from the cluster of “typical aerial hawkers” due to the distinct shape of its papillae. This may reflect the phylogenetic distance between Miniopteridae, to which this species belongs, and vespertilionids (Miller‐Butterworth et al. 2007).

Our analysis confirmed the prediction that typical aerial hawkers possess prominent papillae adorning the forefront of the MDL and pointing forward, specializations that likely serve to block incoming food or non‐food items at high speeds, associated with aerial hawking foraging. The shape of mechanical papillae in bats has been previously suggested to relate to their feeding ecology (e.g. Pastor et al. 1993; Sharma et al. 1999; Masuko et al. 2007; Gregorin and Zanatta 2021). These patterns suggest that the estimated MDL height and papilla shape may facilitate the efficient securing and processing of prey while the bat is flying. This indicates a pathway of integrated morphological specialization, where both behavioral and morphological traits have evolved in tandem to maximize foraging efficiency. The highly specialized MDL in aerial hawkers may have co‐evolved with echolocation capabilities, ensuring precise prey detection and handling even at high speeds.

A tall MDL and prominent papillae are unlikely to be associated with food crushing. *Myotis myotis* primarily feeds on coriaceous insects such as coleopterans (Arlettaz et al. 1997). However, as a substrate gleaner, seizing food from the ground (Arlettaz 1996), this species exhibits a shallow MDL and small papillae that do not point forward, consistent with our interpretation. Rhinolophids emerged as outliers in our analysis due to their very high estimated MDL height values and papilla pointing inward, but more species should be analyzed to confirm this pattern. These bats are evolutionarily distant from other laryngeal echolocators (Teeling et al. 2002; Stoffberg et al. 2010), and their ecomorphology may have been shaped by perch hunting, where jaw morphology is key in targeting prey like moths and beetles (Bogdanowicz et al. 1999). Perch hunting likely influenced rhinolophids’ high‐duty cycle echolocation, allowing them to detect prey while stationary and overcome limitations related to body size and echolocation frequency (Bogdanowicz et al. 1999). Beyond its estimated height, a distinguishing feature of rhinolophids' MDL is its pronounced mobility, potentially linked to processing larger food items from a perch (Voigt et al. 2010). These bats’ unique feeding behavior, combined with their evolutionary history and perhaps their distinct echolocation, likely explains their outlier status. The limited development of the MDL and the absence of prominent papillae in trawling bats is unsurprising, as these bats typically capture prey just above the water surface or directly from it using their feet or wing membranes before bringing it to their mouth (Kalko and Schnitzler 1989). In this capture strategy, prey is tightly controlled, removing the need for specialized tongue structures.

While specialized aerial hawkers form a distinct cluster, other species that use aerial hawking to some extent fell outside the group based on MDL traits. For example, *Myotis emarginatus* and *M. mystacinus* capture prey both in flight and from vegetation surfaces (Dietz and Pir 2021; Budinski and López‐Baucells 2023), lacking the MDL characteristics of specialized aerial hawkers. These “jack of all trades” likely exhibit a more flexible tongue morphology suited to varying foraging strategies. Even species more commonly identified as aerial hawkers, such as *Eptesicus* spp., may occasionally glean prey during brief landings on the ground or in trees (Martinoli et al. 2023). Apart from specialized aerial hawkers, many bats alternate between aerial hawking and gleaning, indicating that rigid classification into these two categories overlooks their complex foraging behaviors.

The case of *B. barbastellus*, a species that hunts prey on the wing using a “stealth echolocation strategy” (Goerlitz et al. 2010), presents an intriguing exception. Despite having a relatively tall MDL, comparable to that of specialized aerial hawkers, the MDL's papillae resemble those of a gleaner. This may be explained by the evolutionary trajectory of stealth echolocation, which evolved from gleaning ancestors rather than from quieting a high‐intensity aerial hawker. Ancestral reconstructions indicate that the Plecotini ancestor used low‐intensity calls typical of gleaners. As barbastelles transitioned to aerial hawking, they retained these low‐intensity nasal calls instead of adopting the high‐intensity echolocation common among other aerial hawkers (Lewanzik et al. 2023).

MDL's diverse morphology among different foraging groups supports the notion that its evolution is shaped by the bat's ecological role rather than by phylogenetic constraints alone. This reinforces the idea that MDL structure may function as an adaptive trait shaped by foraging demands rather than merely a byproduct of dietary habits or phylogenetic history.

Our study has several caveats. First, it is based on only 24 species (covering all European families) out of the over 1000 insectivorous bat species known to science (Simmons 2005), so broad generalizations warrant caution. However, the pattern we identified suggests that MDL morphology may be a predictor of foraging style, even in the absence of other information. Additionally, our study is correlational, as we lack direct evidence of the MDL's role. However, our explanation is plausible, given that debris and food remains are often observed entangled in the spiky papillae of aerial hawkers' MDLs. Furthermore, we conducted our study on live individuals. The notably high mobility of the MDL in rhinolophids may have influenced the accuracy of measurements taken from photographs, potentially leading to variations in observed dimensions, so such measurements must be considered with prudence. While taking measurements from photographs can be challenging, we are confident that the pattern we identified is valid. It is also worth noting that working with threatened mammals like bats presents ethical constraints, which preclude euthanasia in most cases. Observing a muscular structure like the MDL is best done on live individuals, as they retain the muscular tone necessary for its functional assessment. However, future studies might explore high‐tech approaches, such as CT scanning, to determine whether this character remains measurable in museum specimens.

In conclusion, while we do not claim to offer the final word on the MDL's function, we have uncovered a noteworthy pattern that warrants further investigation. Our study is one of the first to quantitatively link MDL morphology to specific foraging strategies across many species. Insectivorous bats exhibit adaptations such as a tall MDL and forward‐pointing mechanical papillae, which help secure food items captured during high‐speed, dynamic foraging. A future direction for research should involve examining a broader range of species, particularly from the many bat families we have not yet covered, including pteropodids, exploring if similar morphological specializations can be identified in non‐European species with comparable foraging strategies. Additionally, analyzing bat species with different feeding specializations will determine if similar morphological trends exist outside the insectivorous guild. Advancing imaging techniques and 3D reconstructions could reveal new insights into how these structures interact with prey during the foraging process. Should the patterns we have described be confirmed across a broader range of species globally, we advocate for including this trait in descriptions of foraging guilds, alongside established traits such as wing shape, echolocation call structure, and emergence time.

## Conflicts of Interest

The authors declare no conflicts of interest.

## Supporting information




**Figure S1** Map representing locations in Portugal and Italy where bats were captured.

**Figure S2** Boxplots representing the median and quartile values of estimated papilla angle, estimated medio dorsal lobe (MDL) height, and forearm length (FAL) for each bat foraging guild.
