# Supplementary figures and images for: Bat Tongues and Foraging: Linking Morphology to Hunting Strategies
Source: Integr Zool. 2025 Apr 9;21(3):540–51. doi: 10.1111/1749-4877.12982 (PMC13165712; doi:10.1111/1749-4877.12982)

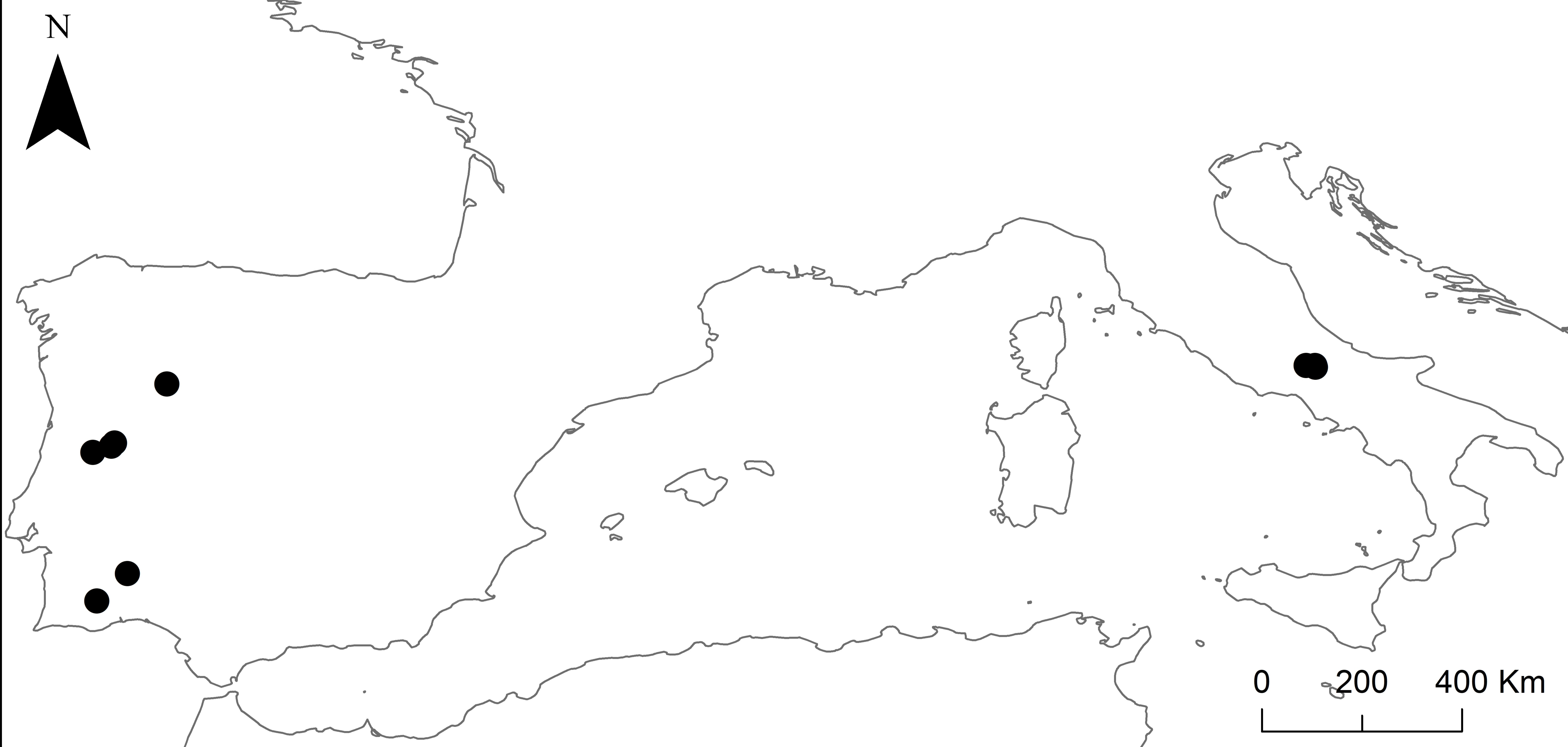

Supplement: Supplementary file 1 — Figure S1 Map representing locations in Portugal and Italy where bats were captured. [file INZ2-21-540-s001.JPG]

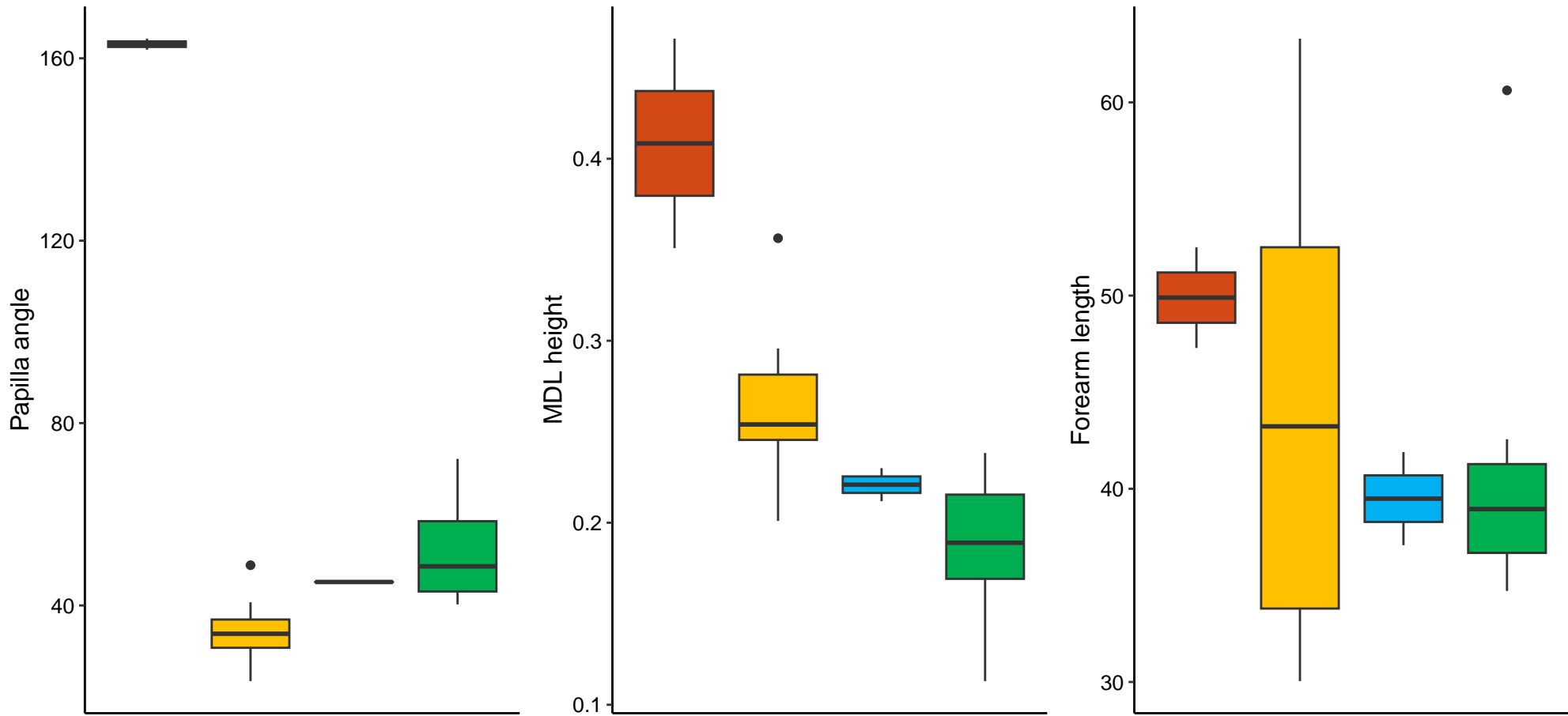

Supplement: Supplementary file 2 — Figure S2 Boxplots representing the median and quartile values of estimated papilla angle, estimated medio dorsal lobe (MDL) height, and forearm length (FAL) for each bat foraging guild. [file INZ2-21-540-s002.pdf]
